# Draft Genome Sequences of Extended-Spectrum β-Lactamase-Producing *Escherichia coli* Strains Isolated from Patients in Lebanon

**DOI:** 10.1128/genomeA.00123-14

**Published:** 2014-02-20

**Authors:** Sima Tokajian, Jonathan A. Eisen, Guillaume Jospin, Anna Farra, David A. Coil

**Affiliations:** a Lebanese American University, Department of Natural Sciences, School of Arts and Sciences, Byblos, Lebanon; b University of California Davis Genome Center, Davis, California, USA; c Lebanese American University, School of Medicine, Byblos, Lebanon

## Abstract

We present the draft genome sequences of nine extended-spectrum β-lactamase (ESBL)-producing *Escherichia coli* strains isolated from stool samples collected from patients admitted for gastrointestinal and urological procedures/surgeries. An average of 3,889,300 paired-end reads per sample were generated, which assembled in 77 to 157 contigs.

## GENOME ANNOUNCEMENT

Resistance to the expanded-spectrum cephalosporins can occur in *Escherichia coli* via the production of extended-spectrum β-lactamases (ESBLs) encoded by a variety of transmissible genes (1). The prevalence of *E. coli* producing ESBLs has increased worldwide during the past decade and has been associated with hospital- and community-acquired infections (2). In this study, we selected 9 ESBL-producing *E. coli* isolates from stools of patients admitted for gastrointestinal and urological procedures/surgeries at the University Medical Center-Rizk Hospital (UMCRH) in Lebanon.

Illumina paired-end libraries were made from sonicated DNA using a TruSeq DNA sample prep version 2 kit (Illumina). Fragments between 300 and 600 bp were selected using a Pippin Prep DNA size selection system (Sage Science). The samples were pooled together and then sequenced on an Illumina MiSeq for paired-end 250-bp reads. An average of 3,889,300 paired-end reads per sample were generated. Quality trimming and error correction of the reads resulted in an average of 3,621,916 high-quality reads. All sequence processing and assembly were performed using the a5 assembly pipeline. This pipeline automates the processes of data cleaning, error correction, contig assembly, scaffolding, and quality control (3). The initial assembly produced the following for each strain: strain LAU-EC2, 119 contigs (no scaffolding); LAU-EC3, 139 contigs contained in 114 scaffolds; LAU-EC4, 157 contigs (no scaffolding); LAU-EC5, 152 contigs contained in 137 scaffolds; LAU-EC6, 106 contigs (no scaffolding); LAU-EC7, 101 contigs contained in 77 scaffolds; LAU-EC8, 109 contigs (no scaffolding); LAU-EC9, 114 contigs (no scaffolding); and LAU-EC10, 147 contigs (no scaffolding). The final draft genome sequences consist of the following: for LAU-EC2, 119 contigs, including a combined 5,183,692 bases with 50.7% G+C content; for LAU-EC3, 139 contigs, including a combined 5,317,089 bases with 50.5% G+C content; for LAU-EC4, 157 contigs, including a combined 5,326,910 bases with 50.6% G+C content; for LAU-EC5, 152 contigs, including a combined 5,317,569 bases with 50.6% G+C content; for LAU-EC6, 106 contigs, including a combined 5,362,824 bases with 50.3% G+C content; for LAU-EC7, 101 contigs, including a combined 5,286,581 bases with 50.7% G+C content; for LAU-EC8, 109 contigs, including a combined 5,243,332 bases with 50.5% G+C content; for LAU-EC9, 114 contigs, including a combined 5,249,256 bases with 50.5% G+C content; and for LAU-EC10, 147 contigs, including a combined 5,300,581 bases with 50.6% G+C content

### Nucleotide sequence accession numbers.

These whole-genome shotgun projects have been deposited at DDBJ/EMBL/GenBank under accession no. AYLY00000000 (LAU-EC2), AYOO00000000 (LAU-EC3), AYOP00000000 (LAU-EC4), AYOG00000000 (LAU-EC5), AYNF00000000 (LAU-EC6), AYNG00000000 (LAU-EC7), AYNH00000000 (LAU-EC8), AYNI00000000 (LAU-EC9), and AYNJ00000000 (LAU-EC10). The versions described in this paper are the first versions, accession no. AYLY01000000, AYOO01000000, AYOP01000000, AYOG01000000, AYNF01000000, AYNG01000000, AYNH01000000, AYNI01000000, and AYNJ01000000, respectively.
